# General and disease-specific health literacy in patients with heart failure: an outcome analysis

**DOI:** 10.1093/eschf/xvag204

**Published:** 2026-07-23

**Authors:** Klara Rebernik Grah, Mitja Lainscak, Jerneja Farkas

**Affiliations:** National Institute of Public Health, Trubarjeva cesta 2, SI-1000 Ljubljana, Slovenia; Faculty of Medicine, University of Ljubljana, Vrazov trg 2, SI-1000 Ljubljana, Slovenia; Faculty of Medicine, University of Ljubljana, Vrazov trg 2, SI-1000 Ljubljana, Slovenia; General Hospital Murska Sobota, Rakičan, Ulica dr. Vrbnjaka 6, SI-9000 Murska Sobota, Slovenia; National Institute of Public Health, Trubarjeva cesta 2, SI-1000 Ljubljana, Slovenia; Faculty of Medicine, University of Ljubljana, Vrazov trg 2, SI-1000 Ljubljana, Slovenia; General Hospital Murska Sobota, Rakičan, Ulica dr. Vrbnjaka 6, SI-9000 Murska Sobota, Slovenia

**Keywords:** Heart failure, Health literacy, Self-care behaviours, Outpatient care, Longitudinal study, Clinical outcome

## Abstract

**Introduction:**

Limited health literacy is common among patients with heart failure (HF), and the relative importance of general and disease-specific health literacy for patient outcomes remains unclear. This study aimed to evaluate general and disease-specific health literacy in patients with HF and to examine their association with behavioural, patient-reported and clinical outcomes.

**Methods:**

This prospective observational study included 126 outpatients with HF (mean age 69 ± 11 years; 71% male) who were followed for 6 months. General health literacy was assessed using the Brief Health Literacy Screen (BHLS), and disease-specific health literacy using the Heart Failure-Specific Health Literacy Scale (HFsHLS).

**Results:**

The two measures were moderately positively correlated (Spearman's ρ = 0.49, *P* < .001), indicating partial overlap. Limited general health literacy (BHLS ≤ 9) was identified in 22% of patients and was associated with lower educational level, more depressive symptoms, higher NT-proBNP levels, and a greater number of prescribed medications (all *P* < .05). In univariable analyses, both health literacy measures were associated with better HF-related knowledge (*P* = .004 and .049), higher health-related quality of life (*P* < .001 and .007), and better self-rated health (*P* < .001 for both), which was not sustained after multivariable adjustment. Additionally, disease-specific health literacy was associated with self-care behaviours (*P* = .033), also after multivariable adjustment (*P* = .026). Neither measure was independently associated with clinical outcomes (death, HF hospitalization, emergency department visit); however, patients with limited general health literacy showed numerically higher event rates and a tendency towards increased risk of clinical outcomes.

**Conclusion:**

In patients with HF, limited general health literacy was observed in 22% of patients. General and disease-specific health literacy measures were not interchangeable, and only disease-specific health literacy showed an independent association with self-care behaviours. A tendency towards less favourable clinical outcomes was observed among patients with limited general health literacy.

## Graphical Abstract

**Graphical Abstract:**
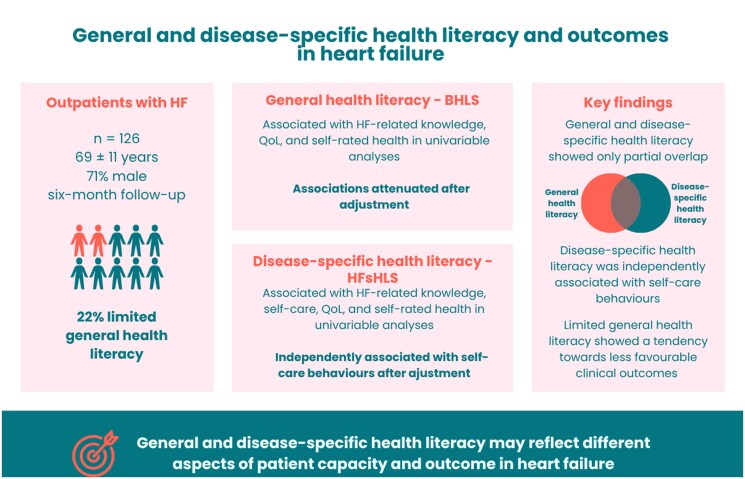

What is new?

- General and disease-specific health literacy in outpatients with heart failure showed only partial overlap, indicating that these measures are not interchangeable and capture different aspects of patient capacity.
- Disease-specific health literacy was independently associated with self-care behaviours, whereas general health literacy showed a consistent tendency towards less favourable clinical outcomes.
- The findings support further evaluation of a staged approach to health literacy assessment in outpatient heart failure care, using general and disease-specific measures as complementary tools to guide patient education and support.

## Introduction

Heart failure (HF) is a complex clinical syndrome affecting ∼26 million people worldwide, with prevalence increasing with age and ranging from 1% to 3% in the general population.^1,2^ Despite advances in diagnostics and treatment, HF remains associated with high morbidity, frequent hospitalizations, and substantial healthcare costs.^3^ In Slovenia, ∼8500 patients are hospitalized annually due to HF,^4^ highlighting the considerable national burden of the disease. As HF is a chronic and progressive condition, optimal management extends beyond medical treatment and requires patients’ active engagement in self-care. Effective self-care includes adherence to prescribed medication, daily weight monitoring, restriction of salt intake, regular physical activity, and timely recognition of worsening symptoms.^5^ Although adequate self-care is associated with improved clinical outcomes,^6^ many patients struggle to perform these behaviours consistently.^7^

Health literacy has emerged as a key factor that may influence patients’ ability to engage in effective self-care.^8,9^ Health literacy refers to the knowledge, motivation, and competencies required to access, understand, appraise, and apply health information in everyday decisions related to health.^10^ In chronic disease management, health literacy can be considered at both a general and a disease-specific level. Whereas general health literacy reflects broader competencies for dealing with health information, disease-specific health literacy refers to competencies related to understanding and managing a particular condition, such as HF.^11^ These two approaches may capture different, although related, aspects of patients’ capacities to manage HF, and may therefore differ in their relevance to specific outcomes.

Over the past decade, health literacy has received increasing attention in research on cardiovascular disease management.^12^ The guidelines of the European Society of Cardiology (ESC) emphasize health literacy as an important strategy to support self-care and improve health outcomes in patients with HF.^2,5^ Limited general health literacy is common among patients with HF and has been associated with poorer disease-related knowledge, worse health status, increased healthcare utilization, and higher mortality.^8,13,14^ However, evidence regarding its association with self-care behaviours remains inconsistent, and most studies in HF have focused on general rather than disease-specific health literacy.^13–15^ As a result, it remains uncertain whether general and HF-specific health literacy are similarly relevant to behavioural and clinical outcomes.

Evidence from other chronic disease populations suggests that disease-specific health literacy measures may be particularly informative for outcomes closely related to day-to-day disease management.^16^ For example, in diabetes mellitus, disease-specific health literacy has been associated mainly with proximal outcomes, such as understanding of care, self-efficacy, communication with healthcare providers, and treatment adherence.^17^ In contrast, associations with hospitalization and mortality have been examined predominantly using general health literacy measures.^18^ Whether a similar pattern applies in HF remains unclear.

This question is particularly relevant in outpatient HF management, where nurses play a central role in patient education, follow-up care, and support for self-care.^19^ A better understanding of the relationship between health literacy, self-care behaviours, and clinical outcomes may therefore help inform more tailored educational approaches and more individualized support in outpatient HF management.^20^ Nevertheless, longitudinal data examining these associations in real-world outpatient settings remain limited,^14^ particularly with regard to the comparative role of general and disease-specific health literacy.

Therefore, the aim of this study was to assess general and disease-specific health literacy in patients with HF and to examine their associations with HF-related knowledge, self-care behaviours, and clinical outcomes, including health-related quality of life, healthcare utilization, and mortality.

## Methods

### Study design

A prospective observational study was conducted to examine general and disease-specific health literacy and their association with behavioural, patient-reported and clinical outcomes in outpatients with HF. The study was conducted at the HF outpatient clinic of General Hospital Murska Sobota, Slovenia. Eligible participants were adults (≥18 years) with a confirmed diagnosis of HF [New York Heart Association (NYHA) functional class II–III] attending their first or a follow-up outpatient visit. Patients with diagnosed dementia or other cognitive impairments that could interfere with their ability to participate were excluded. Sample size was estimated *a priori* using G*Power for the planned multiple regression analyses.^21^ Assuming five predictors, *α* = 0.05, power = 0.80, and a medium effect size, the minimum required sample size was 92 participants. The target sample size was set at ∼120 participants to allow for possible missing data and ensure stable estimates in the adjusted analyses. The study was conducted in accordance with the Declaration of Helsinki and approved by the National Medical Ethics Committee of the Republic of Slovenia (No. 0120-182/2022/3). Written informed consent was obtained from all participants prior to any study-related procedure.

### Measures

Data were collected at baseline, 3 months, and 6 months. At baseline and at 6-month follow-up, all assessments were conducted in person during the outpatient visits. The 3-month follow-up was conducted either during a scheduled clinic visit or via telephone interview if no in-person visit was planned. Participants who did not complete the 3-month assessment remained in the study and were invited to the scheduled 6-month follow-up visit.

At baseline, demographic, socio-economic, and clinical characteristics were collected. Socio-demographic variables included age, gender, marital status, education, employment, and self-assessed economic status. Clinical data comprised NYHA functional class, left ventricular ejection fraction (LVEF), selected laboratory parameters [e.g. N-terminal pro-B-type natriuretic peptide (NT-proBNP), and serum creatinine], comorbidities, and pharmacological treatment extracted from the hospital information system. Depressive symptoms were assessed using the Patient Health Questionnaire-9 (PHQ-9), a nine-item questionnaire with higher scores indicating greater symptom severity.^22^

General health literacy was assessed using the Brief Health Literacy Screen (BHLS), a validated three-item measure designed to identify individuals with limited health literacy.^23^ Disease-specific health literacy was measured using the Heart Failure-Specific Health Literacy Scale (HFsHLS). The HFsHLS evaluates functional, communicative, and critical health literacy across 12 items.^11^ For both scales, higher scores indicated higher health literacy. Completion time is short, with the BHLS requiring ∼1–2 min and the HFsHLS around 5–8 min. Both measures were previously validated for use in the Slovenian language.^24^ Their use enabled the assessment of health literacy both as a general construct and in relation to HF-specific self-management demands.

HF-related knowledge was assessed using the Dutch Heart Failure Knowledge Scale (DHFKS), which consists of 15 multiple-choice items covering general knowledge, treatment-related knowledge, and recognition of symptoms.^25^ Self-care behaviours were measured using the 12-item European Heart Failure Self-care Behaviour Scale (EHFScBS-12). Items are rated on a five-point Likert scale, with higher scores indicating better self-care.^26^

Health-related quality of life and self-rated health were assessed using the EuroQol five-dimension questionnaire (EQ-5D-3L). The measure comprises a descriptive system covering five dimensions (mobility, self-care, usual activities, pain/discomfort, and anxiety/depression), each rated on three levels of severity, and a visual analogue scale (VAS) ranging from 0 (‘worst imaginable health’) to 100 (‘best imaginable health’) to assess overall self-rated health.^27^

Self-monitoring engagement was assessed using a structured HF monitoring diary,^28^ which was provided to all participants at baseline with instructions for daily completion throughout the 6-month period. At the 6-month visit, returned diaries were reviewed by the researcher. A diary entry was defined as one recorded day (one row in the diary) with completed data on body weight, blood pressure, and heart rate. Review of each diary required ∼5–10 min and included verification of entry completeness, counting of recorded days, and assessment of adherence to predefined criteria.

HF-related knowledge, self-care behaviours, health-related quality of life, and self-rated health were reassessed at 3 and 6 months. Data on clinical outcomes including HF-related hospitalizations and emergency department visits due to HF worsening, as well as all-cause mortality, were obtained from medical records at the end of follow-up.

### Statistical analyses

Demographic, socio-economic, and clinical characteristics were summarized using descriptive statistics. Baseline characteristics were compared between health literacy groups using the Mann–Whitney *U* test for continuous variables and the chi-square test for categorical variables. In cases where cell frequencies were too small to meet chi-square assumptions, results were presented descriptively.

General health literacy was operationalised based on the BHLS total score, with participants categorized into limited health literacy (BHLS ≤ 9) and adequate health literacy (BHLS ≥ 10), using a clinically relevant threshold previously applied in HF studies.^29,30^ Disease-specific health literacy was operationalised using the HFsHLS total score. As no established cut-off for this measure is available,^11,31^ a pragmatic threshold was defined based on the BHLS categorization. Patients with HFsHLS scores ≤30 points were classified as having lower disease-specific health literacy, and those with scores ≥31 points as having higher disease-specific health literacy.

The relationship between general health literacy and disease-specific health literacy measures was explored using a scatter plot and Spearman’s rank correlation coefficient, as both health literacy measures showed non-normal distributions (Shapiro-Wilk test, *P* < .001).

Associations of health literacy with behavioural and patient-reported outcomes (HF-related knowledge, health-related quality of life, self-rated health, and HF diary completion) and clinical outcomes (the composite outcome, HF-related hospitalizations, emergency department visits, and death) were analysed using univariable and multivariable regression models, performed separately for general and disease-specific health literacy. Health literacy was analysed as both categorical and continuous variables. Categorical variables were based on predefined or pragmatically defined cut-offs, whereas continuous variables represented total scores of the respective measures. Multivariable models were adjusted for predefined covariates (age, education, NYHA functional class, and depressive symptoms), selected *a priori* based on previous literature and their established clinical relevance as potential confounders of the association between health literacy and HF outcomes.^32–34^

For analysis, diary use was operationalised as a binary variable, with a predefined threshold of at least 84 recorded diary days during the 6-month follow-up period and continued recording at the end of the follow-up. This threshold was pragmatically defined based on the weekly structure of the diary and was intended to reflect sustained and relatively frequent self-monitoring during follow-up. Clinical outcomes were also analysed as binary outcomes.

Longitudinal changes in HF-related knowledge, health-related quality of life, and self-rated health were analysed using linear mixed-effects models. Because self-care behaviours were not assessed at baseline in patients attending their first outpatient visit, this analysis was limited to the 3- and 6-month follow-up assessments.

Time-to-event analyses for clinical outcomes were performed using Kaplan–Meier curves and compared between health literacy groups using the log-rank test. Incidence rates for clinical outcomes were calculated as the number of events per 100 patient-years of follow-up. Cox proportional hazards regression was used to estimate hazard ratios (HRs) with 95% confidence intervals (CI).

Missing questionnaire data were not imputed. Linear mixed-effects models were estimated by maximum likelihood, assuming data were missing at random (MAR). Clinical outcomes were ascertained for all participants throughout follow-up irrespective of questionnaire completion.

All analyses were performed using IBM SPSS Statistics, version 25 (IBM Corp., Armonk, NY, USA). A two-sided *P*-value < .05 was considered statistically significant.

## Results

### Patient characteristics

**Figure 1:**
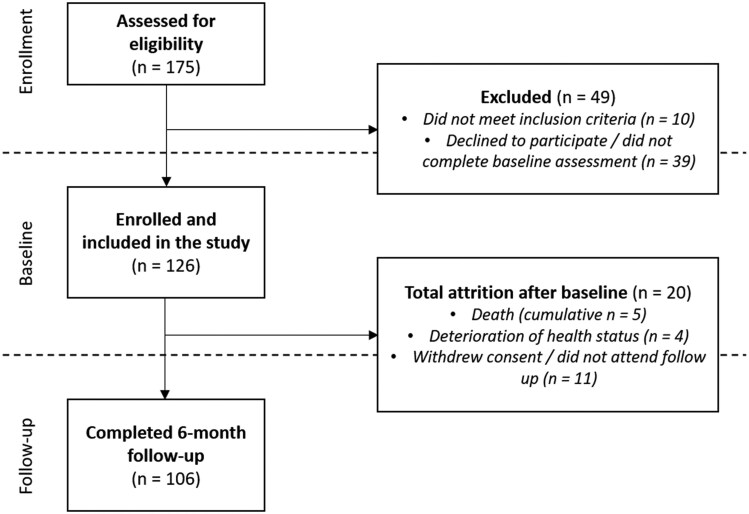
Flow chart of participant recruitment and follow-up.

A total of 175 patients were assessed for eligibility, of whom 126 (72%) were enrolled and included in baseline analyses (*Figure 1*). At 6-month follow-up, data were available for 106 patients (84%). Data at 3 months were available for 73 (58%) participants and were used in longitudinal analyses.

The cohort (mean age 69 ± 11 years; 71% male), was clinically stable, and patients were receiving guideline-directed pharmacological therapy (97% renin-angiotensin-aldosterone system inhibitors, 97% β-blockers, 94% SGLT2 inhibitors). Baseline characteristics overall and according to general and disease-specific health literacy groups are presented in *Table 1*. Participants with limited general health literacy had lower educational level (*P* = .001), more depressive symptoms (*P* = .004), higher NT-proBNP levels (*P* = .046), and a greater number of prescribed medications (*P* = .034) compared with those with adequate health literacy. Similarly, patients with lower disease-specific health literacy had lower educational level (*P* = .035) and more depressive symptoms (*P* < .001) compared with those with higher disease-specific health literacy. No other significant differences in baseline characteristics were observed between the groups. Employment and self-assessed economic status are presented descriptively due to small cell frequencies.

**Table 1:** Baseline characteristics of the study population overall and according to general and disease-specific health literacy groups

Characteristics	Overall	General health literacy (BHLS)			Disease-specific health literacy (HFsHLS)		
	(*n* = 126)	Limited (*n* = 28)	Adequate (*n* = 98)	*P*-value	Lower (*n* = 18)	Higher (*n* = 106)	*P*-value
	*n (%) or mean (SD)*	*n (%) or mean (SD)*	*n (%) or mean (SD)*		*n (%) or mean (SD)*	*n (%) or mean (SD)*	
Age, years	69 (11)	72 (13)	68 (11)	.080	70 (15)	69 (11)	.592
Gender							
Male	90 (71)	20 (71)	70 (71)	1.000	10 (56)	79 (75)	.098
Level of education				.**001**^a^			.**035**
≤Elementary school	38 (30)	**16** (**57)**	**22** (**23)**		**10** (**56)**	**27** (**26)**	
Middle school	75 (60)	**12** (**43)**	**63** (**64)**		**7** (**39)**	**67** (**63)**	
≥High school	13 (10)	**0** (**0)**	**13** (**13)**		**1** (**5)**	**12** (**11)**	
Marital status				.069			.114
Single	13 (10)	6 (21)	7 (7)		4 (22)	8 (7)	
Married/common-law	89 (71)	16 (58)	73 (75)		10 (56)	79 (75)	
Divorced/widowed	24 (19)	6 (21)	18 (18)		4 (22)	19 (18)	
Employment status				—			—
Employed	12 (10)	0 (0)	12 (12)		0 (0)	12 (11)	
Retired	100 (79)	22 (78)	78 (80)		14 (78)	85 (80)	
Unemployed	11 (9)	5 (18)	6 (6)		3 (17)	7 (7)	
Housewife/farmer	3 (2)	1 (4)	2 (2)		1 (5)	2 (2)	
Ease of paying monthly bills				—			—
Very easy	10 (8)	1 (4)	9 (9)		1 (6)	9 (8)	
Easy	61 (48)	9 (32)	52 (53)		6 (33)	55 (52)	
Difficult	44 (35)	13 (46)	31 (32)		8 (44)	36 (34)	
Very difficult	7 (6)	2 (7)	5 (5)		1 (6)	5 (5)	
Non-respondent	4 (3)	3 (11)	1 (1)		2 (11)	1 (1)	
NYHA classification				.137			.147
II	114 (91)	23 (82)	91 (93)		15 (83)	99 (92)	
III	12 (10)	5 (18)	7 (7)		3 (17)	7 (8)	
Depressive symptoms (PHQ-9 score)	3 (4)	**5** (**5)**	**2** (**3)**	.**004**	**6** (**5)**	**2** (**3)**	**<**.**001**
Arterial hypertension	65 (52)	14 (50)	51 (52)	.849	10 (56)	55 (52)	.773
Chronic kidney disease	29 (23)	7 (25)	22 (22)	.777	4 (22)	23 (22)	.960
Diabetes mellitus	32 (25)	9 (32)	23 (24)	.352	4 (22)	27 (26)	.768
LVEF classification				.073			.063
LVEF ≤40%	79 (63)	21 (75)	58 (59)		15 (83)	62 (58)	
LVEF 41–49%	24 (19)	6 (21)	18 (19)		3 (17)	21 (20)	
LVEF ≥50%	23 (18)	1 (4)	22 (22)		0 (0)	23 (22)	
Creatinine, μmol/L	114 (60)	122 (60)	109 (60)	.066	100 (31)	115 (63)	.517
NT-proBNP, ng/L	2038 (2585)	**3307** (**729)**	**1669** (**207)**	.**046**	3365 (4359)	1817 (2130)	.446
Number of prescribed medications	8 (3)	**10** (**4)**	**8** (**3)**	.**034**	8 (4)	8 (3)	.561

Abbreviations: LVEF, left ventricular ejection fraction; NT-proBNP, N-terminal pro-B-type natriuretic peptide; NYHA, New York Heart Association; PHQ-9, Patient Health Questionnaire-9; SD, standard deviation

_a_Bold values indicate statistically significant between-group differences (*P* < 0.05).

### General and disease-specific health literacy

**Figure 2:**
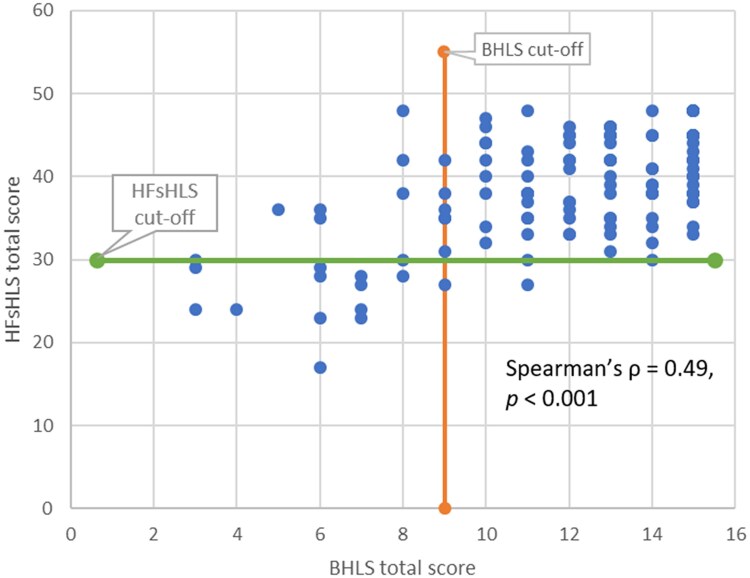
Scatter plot showing the relationship between general health literacy (BHLS) and disease-specific health literacy (HFsHLS).

The mean BHLS score was 12 ± 3 points. Based on the predefined cut-off, 28 participants (22%) were categorized as having limited general health literacy, while 98 participants (78%) had adequate general health literacy. The mean HFsHLS total score was 39 ± 6 points. Based on the pragmatically defined cut-off, 18 participants (15%) were categorized as having lower disease-specific health literacy, while 106 participants (85%) had higher disease-specific health literacy. A moderate positive association was observed between the two measures (Spearman’s *ρ* = 0.49, *P* < .001), indicating only partial overlap between them. Visual inspection of the scatter plot (*Figure 2*) showed substantial individual variability across the score range.

### Association of general and disease-specific health literacy with behavioural and patient-reported outcomes

In univariable analyses, both general and disease-specific health literacy, assessed as categorical and continuous measures, were associated with several behavioural and patient-reported outcomes. After multivariable adjustment, general health literacy was not independently associated with any outcome, whereas disease-specific health literacy remained independently associated with self-care behaviours when analysed as a continuous measure (*B* = 0.28, 95% CI 0.04–0.53; *P* = .026), while no consistent associations were observed when using the categorical approach. No independent associations were observed for HF diary completion (*Table 2*).

**Table 2:** Associations of general and disease-specific health literacy (categorical and continuous) with behavioural and patient-reported outcomes at 6-month follow-up

Outcome	General health literacy (BHLS)				Disease-specific health literacy (HFsHLS)			
	Univariable		Multivariable		Univariable		Multivariable	
*Effect (95% CI)*	Categorical	Continuous	Categorical	Continuous	Categorical	Continuous	Categorical	Continuous
HF-related knowledge	***B*** **=** **1.75 (0.34–3.15), *P*** **=** **.017^a^**	***B*** **=** **0.26 (0.09–0.44), *P*** **=** **.004**	*B* = 0.42 (−1.03–1.87), *P* = .576	*B* = 0.13 (−0.06–0.32), *P* = .191	*B* = 1.253 (−0.35–2.85), *P* = .128	***B*** **=** **0.09 (0.00–0.17), *P*** **=** **.049**	*B* = 0.258 (−1.37–1.89), *P* = .757	*B* = 0.04 (−0.05–0.12), *P* = .401
Self-care behaviours	*B* = 2.68 (−1.16–6.52), *P* = .173	*B* = 0.26 (−0.23–0.75), *P* = .289	*B* = 2.61 (−2.28–6.80), *P* = .332	*B* = 0.27 (−0.33–0.87), *P* = .379	*B* = 2.25 (−2.07–6.57), *P* = .309	***B*** **=** **0.24 (0.02–0.46), *P*** **=** **.033**	*B* = 2.94 (−2.17–8.05), *P* = .263	***B*** **=** **0.28 (0.04–0.53), *P*** **=** **.026**
Health-related quality of life	***B*** **=** **0.26 (0.12–0.40), *P*** **<** **.001**	***B*** **=** **0.03 (0.02–0.05), *P*** **<** **.001**	*B* = 0.07 (−0.06–0.20), *P* = .302	*B* = 0.00 (−0.01–0.02), *P* = .644	***B*** **=** **0.22 (0.05–0.38), *P*** **=** **.013**	***B*** **=** **0.01 (0.00–0.02), *P*** **=** **.007**	*B* = −0.01 (−0.16–0.14), *P* = .865	*B* = 0.00 (−0.01–0.01), *P* = .527
Self-rated health	***B*** **=** **11.35 (2.70–19.99), *P*** **=** **.012**	***B*** **=** **2.09 (1.03–3.14), *P*** **<** **.001**	*B* = 1.04 (−7.57–9.65), *P* = .813	*B* = 0.81 (−0.31–1.93), *P* = .155	***B*** **=** **12.19 (2.15–22.23), *P*** **=** **.019**	***B*** **=** **0.87 (0.36–1.37), *P*** **<** **.001**	*B* = 1.37 (−8.61–11.35), *P* = .788	*B* = 0.39 (−0.09–0.87), *P* = .107
HF diary completion (yes/no)	OR = 1.88 (0.66–5.37), *P* = .243	OR = 1.01 (0.87–1.17), *P* = .887	OR = 1.79 (0.53–6.00), *P* = .343	OR = 1.02 (0.84–1.22), *P* = .869	OR = 2.35 (0.68–8.01), *P* = .174	OR = 1.01 (0.95–1.08), *P* = .741	OR = 4.18 (0.91–19.10), *P* = .066	OR = 1.02 (0.94–1.10). *P* = .677

Abbreviations: BHLS, Brief Health Literacy Screen; CI, confidence interval; HF, heart failure; HFsHLS, Heart Failure-Specific Health Literacy Scale; OR, odds ratio

Note: Values are presented as regression coefficients (B) or odds ratios (OR) with 95% confidence intervals. Multivariable models adjusted for age, educational level, NYHA class, and depressive symptoms.

^a^Bold values indicate statistically significant regression estimates (*P* < 0.05).

In addition, longitudinal analyses were performed to further explore differences in trajectories of behavioural and patient-reported outcomes according to general health literacy groups. Patients with limited general health literacy consistently demonstrated lower HF-related knowledge (*P* = .007), as well as poorer health-related quality of life (*P* = .002) and self-rated health (*P* = .007) compared with those with adequate general health literacy. However no significant effect of time or interaction between time and general health literacy was observed for any of these outcomes. No significant changes or between-group differences were observed for self-care behaviours between 3 and 6 months (Supplementary Figure S1).

### Association of general and disease-specific health literacy with clinical outcomes

In univariable analyses, neither general nor disease-specific health literacy, assessed as categorical or continuous measures, was significantly associated with clinical outcomes. Similarly, no independent associations were observed after multivariable adjustment for the composite outcome (Supplementary Table S1).

**Figure 3:**
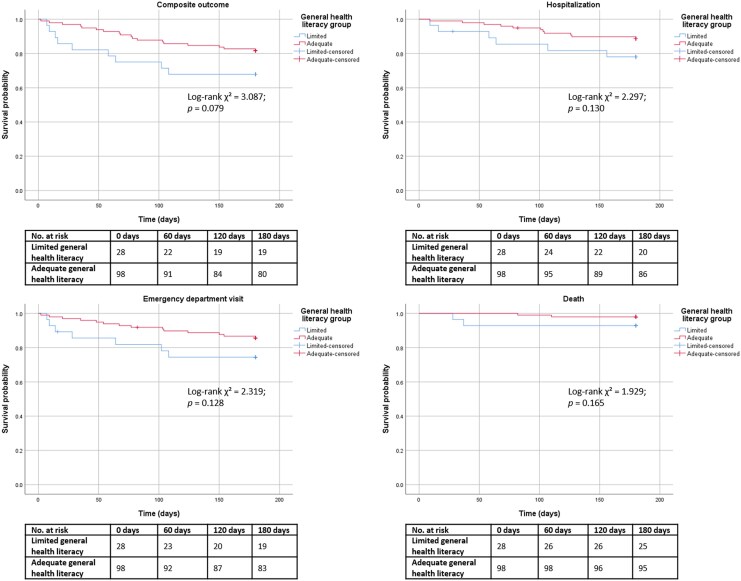
Kaplan–Meier curves for time to composite outcome, HF-related hospitalization, emergency department visit, and death according to general health literacy groups.

**Figure 4:**
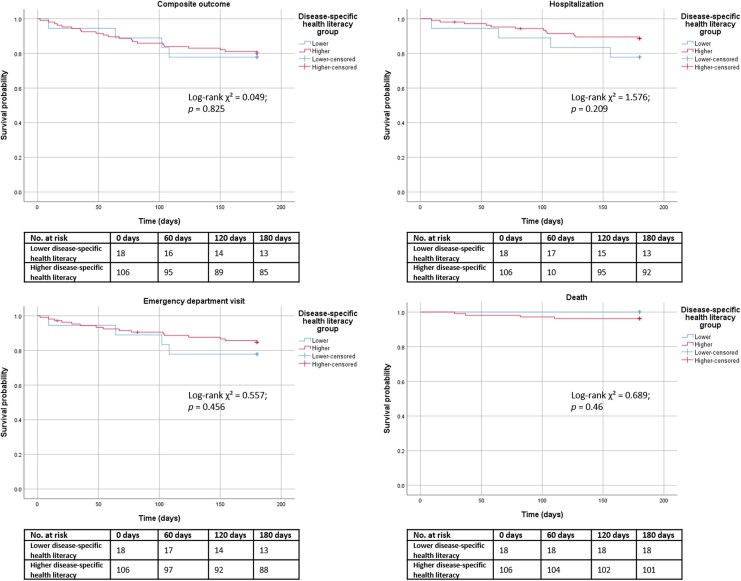
Kaplan–Meier curves for time to composite outcome, HF-related hospitalization, emergency department visit, and death according to disease-specific health literacy groups.

Kaplan–Meier analyses showed no statistically significant differences in time to clinical outcomes according to general health literacy (*Figure 3*). Similar findings were observed for disease-specific health literacy (*Figure 4*).

Patients with limited general health literacy showed consistently higher event rates across all outcomes compared with those with adequate general health literacy. In contrast, disease-specific health literacy showed less consistent differences across outcomes. In Cox regression analyses, limited general health literacy was consistently associated with a higher risk across all outcomes, including the composite outcome, HF-related hospitalization, and emergency department visits, with hazard ratios of approximately two, although these associations did not reach statistical significance. Similar findings were observed after adjustment for covariates for the composite outcome. In contrast, disease-specific health literacy was not associated with the composite outcome and demonstrated weaker and inconsistent associations across individual outcomes (*Table 3*).

**Table 3:** Incidence rates and Cox regression analyses for clinical outcomes according to general and disease-specific health literacy groups

Outcome	General health literacy (BHLS)			Disease-specific health literacy (HFsHLS)		
	Event rates limited vs adequate *Events/100 PY*	Univariable *HR (95% CI)*	Multivariable *HR (95% CI)*	Event rates lower vs higher *Events/100 PY*	Univariable *HR (95% CI)*	Multivariable *HR (95% CI)*
Composite outcome^a^ (*n* = 27)	85.9 vs 41.6	HR = 2.02 (0.91–4.50), *P* = .085	HR = 1.97 (0.75–5.17), *P* = .170	52.1 vs 45.8	HR = 1.13 (0.39–3.29), *P* = .826	HR = 0.78 (0.22–2.73), *P* = .694
Hospitalization (*n* = 17)	51.9 vs 24.3	HR = 2.12 (0.78–5.73), *P* = .139	—	51.1 vs 24.8	HR = 2.03 (0.66–6.30), *P* = .219	—
Emergency department visit (*n* = 21)	64.7 vs 31.6	HR = 2.00 (0.81–4.95), *P* = .136	—	52.1 vs 34.1	HR = 1.51 (0.51–4.53), *P* = .459	—
Death (*n* = 4)	15.4 vs 4.2	HR = 3.66 (0.52–25.96), *P* = .195	—	0.0 vs 7.8^b^	−^c^	—

Abbreviations: BHLS, Brief Health Literacy Screen; CI, confidence interval; HFsHLS, Heart Failure-Specific Health Literacy Scale; HR, hazard ratio; PY, patient years

Note: Values are presented as hazard ratios (HR) with 95% confidence intervals. Multivariable models adjusted for age, educational level, NYHA class, and depressive symptoms.

^a^Composite outcome: hospitalization, emergency department visit, or death.

^b^No deaths occurred in the lower disease-specific health literacy group.

^c^Analysis was not performed because no events occurred in one of the disease-specific health literacy categories.

## Discussion

The present study examined general and disease-specific health literacy among outpatients with HF and their association with behavioural, patient-reported, and clinical outcomes. Approximately one in five participants had limited general health literacy, while a smaller proportion demonstrated lower disease-specific health literacy. The two measures were moderately positively correlated, indicating partial overlap. Disease-specific health literacy was independently associated with self-care behaviours, particularly when analysed as a continuous variable, whereas general health literacy was not. In contrast, neither measure was significantly associated with clinical outcomes. However, patients with limited general health literacy demonstrated higher event rates and a tendency towards increased risk.

In our cohort, patients with limited general health literacy and those with lower disease-specific health literacy had lower educational level and more depressive symptoms. In addition, limited general health literacy was also associated with higher NT-proBNP levels and a greater number of prescribed medications. These findings are consistent with previous HF research showing that limited health literacy tends to cluster with socio-demographic disadvantage and indicators of greater clinical burden. Lower educational level, in particular, has been consistently associated with limited health literacy in HF populations.^30,33^ This may support the interpretation that general health literacy may partly capture broader patient vulnerability.

To our knowledge this is the first study to directly compare general and disease-specific health literacy within the same HF population. A possible explanation for the difference between general and disease-specific health literacy in our analyses lies in the constructs measured by the two measures, which is also consistent with the observed partial overlap. The BHLS is a brief screening tool that primarily captures functional health literacy, focusing on basic abilities to understand and use health information.^23^ Accordingly, associations between BHLS and study outcomes were attenuated after adjustment for educational level, depressive symptoms, and disease severity, suggesting that general health literacy may partly reflect broader social and clinical characteristics.^35^ In contrast, the HFsHLS was developed specifically for patients with HF to capture competencies required for disease management beyond basic functional skills.^11^ Consistent with Nutbeam’s model, it includes communicative and critical dimensions of health literacy, which may be particularly relevant for self-care behaviours requiring symptom monitoring, decision-making, and adaptation of daily routines.^19,36^ This interpretation is supported by previous findings demonstrating independent associations between HF-specific, particularly critical, health literacy and self-care behaviours.^20,37^ However, studies using general health literacy measures have reported inconsistent associations with self-care behaviours,^36,38^ which may reflect differences in the constructs captured by these measures.

In longitudinal analyses, behavioural and patient-reported outcomes remained relatively stable over time, without substantial deterioration or improvement during follow-up. This may reflect the characteristics of our cohort, which consisted predominantly of clinically stable outpatients receiving structured follow-up and guideline-directed therapy in a specialized HF outpatient setting.

During follow-up, 21% of patients had at least one clinical outcome event. Incidence rates were numerically higher in patients with limited general health literacy and, to a smaller extent, also in those with lower disease-specific health literacy. The pattern observed for limited general health literacy was consistent across all clinical outcomes, with a clear trend towards statistical significance, whereas this was not the case for disease-specific health literacy. It is possible that with a larger sample size or longer observational period, statistical significance would have been reached, as previously reported for general health literacy.^30,32,39^ Our results should also be interpreted in light of almost complete adherence to guideline-directed medical therapy, which reduces the number of clinical events and limits the potential for identifying significant risk predictors in this clinically stable outpatient cohort. Although no statistically significant associations were observed, limited general health literacy was consistently associated with higher incidence rates and hazard estimates for the composite outcome, HF-related hospitalization, and emergency department visits. In contrast, disease-specific health literacy showed less consistent associations with clinical outcomes and appeared to be more closely related to behavioural outcomes.

These findings have practical implications for outpatient HF management. Our results suggest that general and disease-specific health literacy should not be regarded as interchangeable, but rather as complementary constructs reflecting different aspects of patient capacity. In our analyses, general health literacy appeared to be more closely related to clinical outcomes than disease-specific health literacy, suggesting that even a brief screening tool such as the BHLS may provide a clinically relevant signal of increased patient vulnerability, which is consistent with previous findings.^39^ At the same time, disease-specific measures, such as the HFsHLS, may provide additional insight into competencies required for day-to-day disease management and may therefore help identify patients who require more targeted support in developing HF self-care skills.^20^

Current recommendations emphasize that patient education and self-care support should be adapted to patients’ individual needs, abilities, and health literacy.^5^ In this context, our findings suggest that a staged approach to health literacy assessment could be considered in routine outpatient care. A brief general health literacy assessment could be performed during the initial outpatient evaluation to identify patients at increased vulnerability, whereas disease-specific health literacy assessment may be reserved for patients with limited general health literacy or those experiencing difficulties with self-care, providing more detailed information to guide targeted educational support. Such an approach may represent a pragmatic compromise between the comprehensive patient assessment and the practical constraints of routine HF outpatient care. Further research, particularly implementation and interventional studies, is needed to determine whether this staged approach improves self-care and clinical outcomes before it can be recommended for routine clinical practice.

### Strengths and limitations

This study has several important strengths. First, its longitudinal design enabled examination of associations between health literacy, self-care behaviours, and subsequent clinical outcomes in an outpatient HF population. Second, the study applied both a general and a disease-specific health literacy measure, allowing assessment of different dimensions of health literacy and providing clinically meaningful insights into HF self-care. The use of a previously validated Slovenian version of the HFsHLS ensured that disease-specific health literacy was assessed using a measure adapted to the local cultural and linguistic context. Data collection within a structured outpatient follow-up programme supported consistent recruitment procedures and high data quality. Furthermore, the study contributes evidence from a Central and South-eastern European setting, where data on health literacy in HF populations remain limited.

Some limitations should also be acknowledged. The study was conducted in a single general hospital, which may limit generalizability; however, the organization of HF care across Slovenian regional hospitals is relatively comparable.^4^ The follow-up period was relatively short and the number of adverse events was low, which may have reduced statistical power and limited the stability of multivariable estimates for clinical outcomes. In addition, participant attrition occurred during follow-up. Finally, the use of self-reported measures may have introduced social desirability bias and may not fully reflect actual self-care behaviours. Group-based analyses for the HFsHLS were based on pragmatically defined cut-off values, as no established thresholds are currently available. This may have limited the comparability of categorized analyses between the two measures.

## Conclusions

In this outpatient HF cohort, limited general health literacy was observed in 22% of patients. General and disease-specific health literacy overlapped only partially and should not be considered interchangeable. While general health literacy was not independently associated with self-care behaviours, it showed a consistent pattern across clinical outcome analyses, with a tendency towards increased risk. In contrast, disease-specific health literacy was independently associated with self-care behaviours. No statistically significant associations were observed with short-term clinical outcomes. These findings suggest that general and disease-specific health literacy capture different but complementary aspects of patient capacity. Disease-specific health literacy may be more informative for understanding and supporting self-care behaviours, while general health literacy may provide a clinically relevant signal of patient vulnerability and could represent a pragmatic target for risk identification in clinical practice. Our findings support further evaluation of a staged approach to health literacy assessment in outpatient HF care, in which general and disease-specific measures are used as complementary tools to guide patient education and support.

## Supplementary Material

xvag204_Supplementary_Data

## Declarations

### Data Availability

The datasets used and/or analyzed during the current study are available from the corresponding author on reasonable request.

### Pre-registered Clinical Trial Number

None supplied.
