# Supplementary material for: General and disease-specific health literacy in patients with heart failure: an outcome analysis
Source: ESC Heart Fail. 2026 Jul 23;13(4):xvag204. doi: 10.1093/eschf/xvag204 (PMC13475125; doi:10.1093/eschf/xvag204)
Supplement: Supplementary file 1 [file xvag204_supplementary_data.docx]

**Supplementary material**

**Figure S1.**

Longitudinal changes in behavioural and patient-reported outcomes according to general health literacy groups.

**Table S1.**

Associations of general and disease-specific health literacy (categorical and continuous) with clinical outcomes at six-month follow-up.

| **Outcome** | **General health literacy (BHLS)** | | | | **Disease-specific health literacy (HFsHLS)** | | | |
| --- | --- | --- | --- | --- | --- | --- | --- | --- |
|  | **Univariable** | | **Multivariable** | | **Univariable** | | **Multivariable** | |
| *Effect (95 % CI)* | Categorical | Continuous | Categorical | Continuous | Categorical | Continuous | Categorical | Continuous |
| Composite outcome (yes/no)* (n = 27) | OR = 0.48 (0.19–1.22), *p* = 0.122 | OR = 0.93 (0.81–1.06), *p* = 0.283 | OR = 0.47 (0.15–1.46), *p* = 0.196 | OR = 0.99 (0.84–1.17), *p* = 0.907 | OR = 0.87 (0.26–2.92), *p* = 0.814 | OR = 0.97 (0.93–1.01), *p* = 0.113 | OR = 1.13 (0.27–4.66), *p* = 0.867 | OR = 0.97 (0.93–1.02), *p* = 0.214 |
| Hospitalization (yes/no) (n = 17) | OR = 0.46 (0.15–1.39), *p* = 0.170 | OR = 0.91 (0.77–1.07), *p* = 0.268 | - | - | OR = 0.45 (0.13–1.57), *p* = 0.211 | OR = 0.98 (0.97–1.00), *p* = 0.119 | - | - |
| Emergency department visit (yes/no) (n = 21) | OR = 0.50 (0.18–1.40), *p* = 0.185 | OR = 0.95 (0.82–1.09), *p* = 0.472 | - | - | OR = 0.62 (0.18–2.14), *p* = 0.450 | OR = 0.99 (0.97–1.01), *p* = 0.184 | - | - |
| Death (yes/no) (n = 4) | OR = 0.27 (0.04–2.01), *p* = 0.202 | OR = 0.86 (0.66–1.10), *p* = 0.229 | - | - | **-**** | OR = 1.02 (0.92–1.13), *p* = 0.748 | - | - |

*Abbreviations: BHLS – Brief Health Literacy Screen; CI – confidence interval; HF – heart failure; HFsHLS – Heart Failure-Specific Health Literacy Scale; OR – odds ratio.*

*Note: Values are presented as odds ratios (OR) with 95% confidence intervals. Multivariable models adjusted for age, educational level, NYHA class, and depressive symptoms. Clinical outcomes (hospitalization, emergency department visit, mortality) were not included in multivariable modeling due to low event frequency.*

**Composite outcome: hospitalization, emergency department visit, or death.*

***Analysis was not performed because no events occurred in one of the disease-specific health literacy categories.*
